# Round the Hospitals

**Published:** 1921-04-09

**Authors:** 


### ROUND THE HOSPITALS.

So long as the headmistresses misunderstand the aims of hospital training nursing will not appeal to girls on leaving school as a possible career; and the College of Nursing has done good service in bringing about an exchange of opinion with representatives of the Headmistresses' Association. The practical outcome of the meeting is the acceptance of an invitation to some of the headmistresses to come and see for themselves what kind of course the leading training-schools for nurses offer to probationers. The College Council have learned for their part how outsiders view the nursing profession as a career. The lack of any stable prospect, whether of rise in salary or superannuation at the close, is one of the main complaints, and of this nurses are fully as well aware as their critics. It is well understood that headmistresses excessively dislike the lack of any kind of educational standard for entrants. The assumption that "any fool is good enough to be a nurse" is distinctly bad all round, especially as in reality probationers are very rigidly tested in many directions.

**ROUND THE HOSPITALS**—(*continued*).

That the importance of raising the standard of knowledge in the nursing profession is recognised by those responsible under State Registration the Headmistresses Association can assure themselves by a perusal of the draft syllabus of lectures and demonstrations for education and training in general nursing which has been compiled and issued by the General Nursing Council, as described in these columns on March 19, page 567. The matrons of training-schools and representatives of nurses' organisations are now considering this syllabus, and at the informal conference to be held on April 28, to which all have been invited, the subject will be fully discussed. The conference promises to be of the greatest value. It has been divided into two parts: (*a*) "The General Register," introduced by Mr. J. C. Priestley, K.C., Chairman of the General Nursing Council; Miss Lloyd Still will speak on the syllabus and examinations, Miss Dowbiggin and Dr. Goodall on the Poor-law infirmary and medical aspects respectively, whilst Miss Cox Davies will sum up; and (*b*) "Reciprocal Training" when such representative authorities as Miss Sparshott, Dr. Bedford Pierce, Miss Seymour Yapp, Miss Villiers and a children's hospital matron will speak on their respective specialities; Mrs. Bedford Fenwick will sum up. We hope the attendance at the conference will be large, so that the opportunity to get to the bottom of this highly important subject may be fully availed of. The press will not, we understand, be admitted to the conferences.

---

It is noteworthy that despite the alleged desire on the part of nurses to be represented by nurses as opposed to matrons, in the case of the five nominations (see p. vi) for the College Council elections by the members of the London Centre (numbering upwards of 850), although two of the nine names presented in the postal ballot were those of nurses, three of the ladies nominated are matrons, one (Lady Cowdray) is not a nurse, while Miss Hughes is the late lady superintendent of Queen Victoria's Jubilee Institute for Nurses. Is this action of the London Centre to be taken as an indication of what will happen when the actual voting for the new Council members takes place? We are glad to see that varying interests are represented by the nominated five, *i.e.*, district nursing by Miss Hughes, small and special hospitals by Miss Redl, the West country and the geographically isolated nurse by Miss Billing, and Poor-law by Miss Masters, while the general welfare of the profession is represented by Lady Cowdray. Other centres have also nominated candidates, who are now, we hope, preparing as in East Lancashire, to address their constituents, so that the election promises to be an interesting one.

---

Sir Cooper Perry, M.D., is to take the chair at the annual meeting of the Chartered Society of Massage and Medical Gymnastics, to be held on April 16 at 3 p.m. in the Armitage Hall, 228 Great Portland Street, W., and will present the first report of the Council, dealing with the work of the Society since it obtained its Royal Charter last year. The meeting promises to be of exceptional interest, the subjects for discussion embracing the special insurance scheme, a proposed club for professional women, and the possible extension of treatment at Red Cross clinics to civilian patients. A design for a coat-of-arms, suitable for a badge of membership, has been approved. A social gathering will be held after the meeting at Mortimer Hall, 93 Mortimer Street, W.

---

We are asked to state that owing to the reduction in train services which has taken place in consequence of the situation created by the impasse between the coalowners and the miners, the quarterly meeting of the Association of Hospital Matrons, which was to have been held at Leeds on Saturday, April 9, has been postponed. Members are asked to note this, as time does not permit of them being notified individually.

---

The history of St. Thomas's Hospital, by the Secretary, Mr. G. Q. Roberts, C.B.E., now appearing in St. Thomas's Hospital *Gazette*, has reached the period 1711. At this epoch it was undergoing reconstruction, and had 556 patients under treatment. Extracts are given from the accounts, from which we learn that the rate of remuneration to the nursing staff was as follows: "Two sisters of the foul wards, £40 each; two nurses of the foul wards, £20 each; two sisters of the salivating wards, £30 each; two nurses of the salivating wards, £18 each; fifteen sisters in the other fifteen wards, £25 each; fifteen nurses in the other fifteen wards, £16 each. A day-watch of Susannah's Ward, 4s. per week; eighteen night-watches of the wards, £10 8s. each per annum." It would seem no board was provided, for the matron was allowed 1s. 6d. per week for board wages and the sisters at the rate of 1s. 4d. per week. Patients' diet cost 2d. per week.

---

In our issue of February 26 we were able to announce that the Queen's Hospital for Children had sanctioned an increased scale of salaries for its nursing staff, which was substantially the same as that recommended by the representatives of the principal London Children's Hospitals meeting in conference. A practically similar scale of increases has now been adopted by the Hospital for Sick Children, Great Ormond Street, and it is retrospective to January 1, 1921. Under it the Matron receives £275, rising to £350; the Assistant Matron £100, rising to £140; Home Sister £90, rising to £130; Night Sister £85, rising to £125; the annual increment in all the foregoing cases is £10. Ward Sisters start at £80 and rise to £120, and staff nurses at £50, rising to £60; the annual increment in each of these two classes being £5. The salaries of probationers under the new scale are £20, £25, and £30.

---

Why is it that some Nursing Associations can secure a good succession of candidates for training and others conspicuously fail? It cannot be alto-

**ROUND THE HOSPITALS**—(*continued*).

gether a question of locality. The Worcestershire Nursing Association, for instance, was able to report at the recent annual meeting, presided over by Lady Georgina Vernon, that the work of the Training Home was increasing. Twenty-two nurses passed the C.M.B. Examination, six candidates were prepared for the Queen's Roll and were admitted into Class A. The salaries of all nurses were raised; seventy nurses are at work in the city and county, and the County Council gratefully acknowledges the assistance rendered during the year. On the other hand, the North Wales Nursing Association reports a great dearth of candidates for training, coupled with the failure of pupils in examinations and the breaking of their agreements by many nurses. An attempt to establish a joint training-centre for North and South Wales has proved abortive. The need for nurses is urgent, but it is impossible in many localities to supply them.

The retirement from the Bristol Royal Infirmary of the two sisters, Miss Fanny and Miss Julia Gross, after thirty-three years' unbroken service, is a very interesting event, typical of a passing generation. During the entire time which they spent together in the hospital, starting as probationers on St. Patrick's Day, 1888, Sister Julia has never been off duty from illness for a single day, and Sister Fanny only one week. This must surely establish a record. A delightful portrait of them is given in the *Western Press*. The Royal Infirmary may well be proud of their loving service so loyally and efficiently rendered for a third of a century.

The nursing staff of the London Temperance Hospital is nobly taking a share in the task of raising funds to build the new and much-needed nurses' home. If the necessary funds are raised, it will be possible to open another ward, a result much to be desired. With this object the nursing staff is giving a musical and dramatic entertainment in the out-patient hall of the hospital on three nights next week—viz. April 13, 14, and 16—for which Miss Donaldson, the energetic matron, is busy selling tickets, priced at 10s. 6d., 5s., 2s. 6d., and 1s. We hope there will be a great demand for these tickets this week, with record attendances, to show the public's appreciation of the efforts of this enterprising nursing staff.

St. Leonard's House, West Malling, has recently been opened as the Frederick Andrew Convalescent Home, under medical direction, for gentlewomen earning their own living, governesses, teachers, artists, authors, journalists, and trained hospital nurses. Mr. Frederic Andrew, a solicitor of good standing, left the bulk of his fortune to trustees to establish and endow this institution. Being a lover of the antique, he directed that the Home should be furnished with historical accuracy in various styles. The result is exceedingly unusual in a home of this description. From Tudor to late Georgian each room is kept true to date, and there are numerous good paintings, engravings and prints. The house is surrounded by seventy-nine acres of park-land, orchards and gardens, and has a winter garden, a well-stocked library, and a music-room.

Volunteer nurses are said to be coming forward well in New York to undertake domestic duties in the hospitals in the scarcity of manual workers. Many of the hospitals have closed half their beds, and a rally from the Red Cross has touched the sympathies of the V.A.D.s who gave their services in the war. The labour shortage in this country has not brought us to the pitch of setting fashionable women to scrub floors and sort the dirty linen, but good organisation could find many more useful services for Red Cross workers than are at present attempted. There is no denying that English hospitals are averse from invoking the V.A.D.

The low salaries, £55 a year only, paid to their nurses by the Cambridge and District Nursing Association occasioned remonstrance from the Chairman, Councillor G. P. Hawkins, on the occasion of the annual meeting. The Corporation pays its very lowest men nearly £200 a year, and Miss Webber contended that if scavengers, whose work is in the open air, are paid at high rates, the nurses, who are expected to encounter grave risks in very stuffy rooms, ought in reason to receive more consideration. There is no question that the nurses' salaries ought at once to be raised, and if Cambridge cannot afford this the Association can at least avoid employing women it cannot afford to pay by reducing its staff.

Mrs. Harwood, a widow who kept lodgings in Taunton, has testified her gratitude for past acts of kindness by leaving all her savings to be divided between the Taunton and Somerset Hospital and the Taunton Workhouse, where her mother was taken care of for some long time. The money for the workhouse, amounting to about £150, is to be invested, and the interest used as a little fund for providing luxuries for the inmates, and especially the women and children, at Christmas.

At Harrow the work of the district nurses is co-ordinated with that of the Cottage Hospital, and at the annual meeting, at which Mr. Macandrew presided, both reports were presented. The part-time services of all the nurses have been secured as health visitors, by the payment of a subsidy of £230 from the urban district council. This is an admirable arrangement, tending to abolish any loophole for friction and save the nurses' valuable time. There are now three infants' clinics at work, with a valuable band of voluntary workers. The hospital is fortunate in the gratuitous and valuable services rendered by four of its nurses, and records its appreciation of Sister Peake's administration.

